# *SmDXS5*, acting as a molecular valve, plays a key regulatory role in the primary and secondary metabolism of tanshinones in *Salvia miltiorrhiza*

**DOI:** 10.3389/fpls.2022.1043761

**Published:** 2022-11-10

**Authors:** Da-chuan Zhang, Ling-long Luo, Zhi-rong Wang, Wen-juan Xu, Jun-ling Li, Shu-ting Tan, Jia-hui Wu, Yan Li, Chi Zhang, Chen Liang, Xue-yong Wang

**Affiliations:** School of Chinese Meteria Medica, Beijing University of Chinese Medicine, Beijing, China

**Keywords:** *Salvia miltiorrhiza*, transcriptome, metabolome, tanshinone, secondary metabolites

## Abstract

Red sage, the dry root and rhizome of the herbaceous plant *Salvia miltiorrhiza* Bunge, is widely used for treating various diseases. The low content of tanshinones (terpenoids) has always restricted development of the *S. miltiorrhiza* industry. Here, we found that *SmDXS5*, a rate-limiting enzyme-coding gene located at the intersection of primary and secondary metabolism, can effectively change the transcription level and secondary metabolome profile of hairy roots of *S. miltiorrhiza*, and significantly increase the content of tanshinones. *Agrobacterium rhizogenes* was used to infuse *S. miltiorrhiza* explants, and hairy roots of *S. miltiorrhiza* expressing the *SmDXS5* gene were obtained successfully. We identified 39 differentially accumulated metabolites (DAMs) by metabolomics based on ultra-high performance liquid chromatography quadrupole exactive mass spectrometry and multivariate statistics. These DAMs might be key metabolites of *SmDXS5* gene regulation. RNA sequencing was used to compare gene expression between the hairy roots of the *SmDXS5* overexpressing group and the blank control (BC) group. Compared with the BC group, 18,646 differentially expressed genes were obtained: 8994 were upregulated and 9,652 downregulated. The combined transcriptome and metabolome analyses revealed that the mevalonate and methylerythritol phosphate pathways and synthase gene expression levels in the *SmDXS5* overexpressing group were upregulated significantly, and the accumulation of tanshinone components was increased significantly, which promoted the process of glycolysis and promoted the transformation of carbohydrates to secondary metabolism. Moreover, the expression of *SmPAL*, the first rate-limiting enzyme gene of the phenylpropane pathway, decreased, reducing the accumulation of phenolic acid, another secondary metabolite. Therefore, *SmDXS5* can be defined as a ‘valve’ gene, mainly responsible for regulating the distribution of primary and secondary metabolic flow of tanshinones in *S. miltiorrhiza*, and for other secondary metabolic pathways. The discovery of *SmDXS5* and its molecular valve function in regulating primary and secondary metabolism will provide a basis for the industrial production of tanshinone components, and cultivation of high quality *S. miltiorrhiza*.

## Introduction

*Salvia miltiorrhiza* Bunge. belongs to the family Labiatae, which has a very high medicinal development value. Its dry roots and rhizomes, red sage (called Danshen in Chinese) has long been used in the famous traditional Chinese medicine (TCM) recorded in the “Chinese Pharmacopoeia”. *S. miltiorrhiza* alone or in combination with other traditional Chinese medicine has a curative effect. *S. miltiorrhiza* is used to treat various diseases, especially cardiovascular diseases (Wang et al., 2017), diabetes (Jia et al., 2019), coronary heart diseases, and cerebrovascular diseases (Su et al., 2015). Chemical constituents of *S. miltiorrhiza* have become a major focus in the related field. The bioactive components of *S. miltiorrhiza* consisted of two main groups, phenolic acids (water-soluble) and tanshinone (lipid-soluble) (Kai et al., 2011). Due to the low content of tanshinone, long growth cycle, and serious quality degradation, supplementation of tanshinone has become a research spotlight in order to meet the increasing demand for clinical applications.

Tanshinones belong to diterpenoids, which are a large group of biologically active compounds (Lois et al., 2000). Tanshinones accumulate in the stems, leaves, flowers, and most notably the roots of *S. miltiorrhiza*, synthesized from common C5 precursors, isopentenyl pyrophosphate, and its isomer dimethylallyl diphosphate (Xu et al., 2010; Xiuchun and Jianyong, 2005; Kai et al., 2011). Over 40 tanshinones and structurally related compounds have been identified in Danshen (Meim et al., 2019).

The metabolic pathway of tanshinone conforms to the rule of the diterpenoid metabolic pathway. Acetyl-CoA plays an important role in the tricarboxylic acid cycle (TCA cycle), fat metabolism, and energy metabolism in primary metabolism, and acts as the “starting substrate” of the mevalonate (MVA) pathway of terpenoid secondary metabolism. Acetyl-CoA regulates secondary metabolism and primary metabolism independently to some extent. It can be said that acetyl-CoA is an important “metabolic button” of the primary and secondary metabolism of terpenoids (Dai et al., 2010; Dai et al., 2010). The biosynthesis of 1-deoxyxylulose 5-phosphate synthases (DXS) is the initial step in the methylerythritol phosphate (MEP) pathway of terpenoids secondary metabolism, and is also the key “step” for pyruvate, the core product of sugar metabolism, to enter the secondary metabolic pathway. Therefore, 1-deoxyxylose-5-phosphate synthase (DXS) is a molecular switch that connects the MEP metabolic pathway (secondary metabolism) with the glucose metabolic pathway (primary metabolism).

The biosynthesis of tanshinones is a complicated process, roughly divided into 3 stages. In the first stage, MEP is the main pathway, supplemented by the mevalonate (MVA) pathway to generate isopentenyl diphosphate (IPP). In the second stage, IPP was transformed into geranyl diphosphate (GPP), farnesyl diphosphate (FPP) and geranylgeranyl diphosphate (GGPP). Finally, GGPP generates diterpenoids through stepwise ionization and cycloisomerization reactions (Pu et al., 2021). All MEP pathway enzymes are located in plastids (Reumann et al., 2007). MEP pathway supplies precursors for plastidial isoprenoid biosynthesis (Wright et al., 2014). Most plant genomes encode multiple DXS isoforms, which belong to distinct phylogenetic groups, types I and II (Walter et al., 2002). Previous studies have shown that type II clade DXS is frequently associated with terpenoid secondary metabolite biosynthesis (Kim et al., 2006; Montserrat et al., 2014). Recent studies revealed the presence of the type III clade (Cordoba et al., 2011). D‐glyceraldehyde 3‐phosphate (GAP) is involved in many metabolic pathways in organisms, such as the glycolysis pathway, gluconeogenesis process, etc. GAP is not only the intermediate product of glycolysis, but also the intermediate product of glycerol isomerization to sugar. It is a chemical substance related to lipids and sugars. Therefore, DXS may play an important role in connecting the MEP pathway and plant primary metabolism. DXS is encoded by a small gene family of five members in *S. miltiorrhiza*, and all 5 genes encode proteins with domains and motifs conserved among previously known DXSs Ma et al., 2012). *SmDXS5* is mainly expressed in leaves and stems, and its expression level is lower compared with the other four *SmDXS* genes.

Previous studies (Ma et al., 2012) showed that the secondary metabolite components of *S. miltiorrhiza* were mainly accumulated in its root, and *SmDXS2* was highly expressed compared with other 4 *SmDXS* genes, indicating the importance of *SmDXS2* in tanshinone synthesis. It is generally believed that the content of secondary metabolites in plants is affected by the gene expression. In our previous experiment, we overexpressed the gene *SmDXS2* with high expression in hairy roots, and the content of tanshinone components in overexpressed *SmDXS2* hairy roots was lower than that in the hairy roots of overexpressed *SmDXS5* gene constructed in this experiment. *SmDXS5* significantly promoted the accumulation of secondary metabolites in hairy roots of *S. miltiorrhiza* compared with *SmDXS2*. This indicates that gene expression level is a factor affecting plant metabolism, but the key factor lies in the response effect of genes. It also confirms the valve role of *SmDXS5* gene and overturns the previous cognition.

*Agrobacterium rhizogenes* is a gram-negative bacterium. It transforms plants by transferring a discrete segment of its DNA, the T-DNA, to plant cells (Kregten et al., 2016). After infecting plants, it will grow a large number of white hairy roots from the wounds of the infected parts. Compared with the roots of the same plant itself, hairy roots have many characteristics, such as rapid growth, short culture period and strong ability to synthesize secondary metabolites, so they can grow rapidly on solid or liquid medium and can accumulate a large number of economically valuable secondary metabolites, so hairy roots are used as good targets for obtaining a lot of plant secondary metabolites (Moyano et al., 2002; Maistrenko et al., 2015). Hairy roots are essentially the product of single cell cloning. They can grow rapidly on hormone-free medium, and their physiological and genetic characteristics can remain stable after repeated subculture. Hairy root culture has developed into a new plant culture system after cell culture. More than 30% of the medicinal parts of medicinal plants are roots, so hairy root culture is very important for investigating the mechanism of secondary metabolite synthesis in medicinal use.

In this study, the hairy root system of transgenic *S. miltiorrhiza* was successfully established, and the *SmDXS5* gene was overexpressed, so as to establish the valve gene research model. Based on metabonomics and transcriptomics methods, the differences in the accumulation of primary products regulated by valve gene and the expression of key genes in biological synthesis pathway were systematically analyzed. It was further verified that the *SmDXS5* gene plays a pivotal and valve role between primary and secondary metabolism in plants. The discovery of valve gene *SmDXS5* laid a foundation for the future research on the regulation mechanism of target genes and the directional cultivation of *S. miltiorrhiza*.

## Materials and methods

### Construction of plant expression vectors

The complete *SmDXS5* (Genbank accession number: JN831118.1) cDNA was isolated and cloned from the sterile seedlings of *S. miltiorrhiza* (Pan et al., 2009; Kai et al., 2010). The vector pCAMBIA1305.2 (CAMBIA) were double-digested with NcoI and PmlI, under the control of the strong cauliflower mosaic virus (CaMV) 35S promoter and NOS terminator to generate a pCAMBIA1305.2-*SmDXS5* construct. The blank vector pCAMBIA1305.2 without exogenous gene was used as the blank control.

### Transformation of *S. miltiorrhiza* and hairy root cultivation

*S. miltiorrhiza* plants were cultivated on Murashige and Skoog (MS) medium at 25 °C under a 16 h light/8 h dark photoperiod, with 0.7 % agar and 3% sucrose (pH 5.8 ± 0.1). All the constructs were introduced into *Agrobacterium tumefaciens* strain C58C1, which were then transformed into *S. miltiorrhiza* to produce transgenic lines with hairy roots. The blank construct (blank vector pCAMBIA1305.2 without exogenous gene) was used as the blank control. The sterile leaves of *S. miltiorrhiza* were taken, the upper and edge parts with thin veins were cut off, submerged in the bacterial suspension for 10-12 min. Then we removed the leaves, dry the bacterial solution with sterile filter paper and then transferred the leaves in MS medium supplemented with 30% sucrose, 0.8% agar, at 22°C in darkness. After co-cultivation for 3 days, the leaves were washed with 60 ml sterilized water three times, and blot-dried on sterile filter paper then transferred to half-strength MS medium with 200 mg/L timentin for 2–3 weeks, in order to eliminate the agrobacteria. Pieces of the hairy roots of approximately 2–3 cm were cut off and transferred to a new MS medium containing timentin. The medium was changed every 7 days, and the concentration of timentin was gradually reduced until there was no plaque. Rapidly growing hairy roots on MS medium were selected and DNA was extracted using plant genomic DNA Extraction Kit (Tiangen). The PCR-positive hairy roots (3-4 cm in length) were then placed in 150 mL erlenmeyer flasks with 100 mL of 6,7-V liquid medium, and grown at 25 °C on an orbital shaker set at 100 rpm in darkness.

### Sample preparation and extraction

Hairy roots samples were freeze-dried and crushed using a mixer mill. Each sample was accurately weighed at 10 mg and placed into a 2 ml centrifuge tube, added 1 ml of 75% methanol and weighed, ultrasonically extracted for 30 min, made up to the weight with 75% methanol, centrifuged at 12000 rpm for 2 min, and the supernatant was absorbed and filtrated (0.22 µm pore size) and filled into liquid-phase vials for testing as hairy roots.

### UPLC-Q-exactive-MS conditions

Hairy roots sample extracts were analyzed based on the UPLC-Q-Exactive-MS system. the UPLC column was a Water CORTECS C18 (2.1×100 mm, 1.6µm). Mobile phase consisted of solvent A was pure water with 0.1% acetic acid while solvent B was pure acetonitrile. Linear gradient programmed as follow: 0.0–3.0 min, 95-5%; 3.0–15.0 min, 80-20%; 15.0–16.0 min, 70-30%; 16.0-27.0 min, 60-40%; 27.0-31 min, 50-50%; 31-34 min, 30-70%; 34-41.0 min, again 90-10% of solvent B. Flow rate: 0.2 ml/min, injection volume: 2 μl. The column temperature was 35 °C and sample manager temperature was 4 °C.

Mass spectrometry conditions: positive and negative ion scanning mode, range of 100-1500 m/z, HESI ion source, ion source temperature 350°C, capillary temperature 320°C, gasification temperature 400°C, spray voltage 3.5 kV, capillary voltage 35 V, tube lens voltage 110 V, sheath gas and auxiliary gas are high purity nitrogen (purity > 99.99%), sheath gas flow rate 35 arb, auxiliary gas flow rate 10 arb, S-lens RF level 55%.

### Identification of metabolites and multivariate statistical analysis

Raw data were converted into mzML format by MSConvert software (https://proteowizard.sourceforge.io/) and uploaded to Xcms Online (https://xcmsonline.scripps.edu). The website performed pre-processing operations such as peak identification, peak correction and finally exported as an xls. format file. The data were imported into MetaboAnalyst 5.0 (https://www.metaboanalyst.ca/) for missing values imputation, denoising and area normalization and cluster analysis.

Multivariate principal component analysis (PCA) and orthogonal partial least squares-discriminant analysis (OPLS-DA) were conducted using the base package and “MetaboAnalystR” in R. Metabolite P-values were calculated by SPSS 19.0, and metabolites that met the conditions of P-value < 0.05 and VIP value > 1 were considered as potential biomarkers. Retrieval of the database HMDB (http://www.hmdb.ca/), METLIN (http://metlin.scripps.edu/) and KEGG (https://www.genome.jp/kegg/pathway.html), compounds were identified and metabolic pathways were analyzed to elucidate their metabolic regulatory mechanisms.

### RNA-seq and functional annotation

Illumina TruseqTM RNA Sample Prep Kit was used for library construction in sequencing experiments. Total RNA was extracted from tissue samples. Nanodrop 2000 was used to detect the concentration and purity of the extracted RNA, agarose gel electrophoresis was used to detect the integrity of RNA, and Agilent 2100 was used to determine the RIN value. Transcriptome sequencing using the Illumina Novaseq 6000 sequencing platform. Based on the existing reference genome of *S. miltiorrhiza*, the software Cufflinks was used (http://cole-trapnelllab.github.io/cufflinks/) and StringTie (http://ccb.jhu.edu/software/stringtie/). The mapped reads were assembled and spliced, compared with the known transcripts, the transcripts without annotation information were obtained, and the potential new transcripts were functionally annotated. In order to obtain more comprehensive gene function information, compare all genes and transcripts obtained by transcriptome assembly with six databases, namely, the Kyoto Encyclopedia of Genes and Genomes (KEGG) (Minoru et al., 2004; Kanehisa et al., 2006), the Nonredundant Protein Database (NR), SwissProt (Magrane, 2011), Gene Ontology (GO) (Ashburner et al., 2000), Pfam, and EggNOG. Comprehensively obtained the functional information of transcripts, and made statistics on the annotation of each database.

The gene expression level was normalized using the FPKM (fragments per kilobase of transcript per million mapped reads) method. After obtaining the read counts of genes/transcripts, the samples that met the criteria of *P*-adjust < 0.05 and |log2 FC ≥ 1| were analyzed for differential expression among samples to identify the differentially expressed transcript information among samples, and then to investigate the function of the differentially expressed genes (DEGs)/transcripts. The major DEGs were clustered using the MetaboAnalyst 5.0 (https://www.metaboanalyst.ca/).

### Confirmation of transcriptome data using quantitative real-time PCR analysis

6 candidate initiation synthase genes linking primary metabolism with secondary terpenoid and phenylpropanoid pathways of *S. miltiorrhiza* were selected to investigate the expression profiles by qRT-PCR analysis, including *SmAACT1*, *SmDXS2*, *SmDXS5*, *SmKSL1*, *SmPAL1* and *SmTAT1*. Transcript-specific primers and gene information were listed in **Table S1**. *SmActin* (F: GGTGCCCTGAGGTCCTGTT; R: AGGAACCACCGATCCAGACA) was selected as an internal control.

The first-strand cDNA was synthesized using the Evo M-MLV RT Premix for qPCR (Accurate Biotechnology, China). Real-time PCR was performed with SYBR Premix Ex Taq II (Takara, Japan). Template cDNA was used for qRT-PCR amplification of genes, and the relative expression level of each gene was detected. Each sample was parallel for 3 times. Ct value obtained from detection was used to calculate the relative expression level of genes by 2^–ΔΔCT^ method (Livak and Schmittgen, 2001).

### Correlation analysis between transcriptome and metabolome data

Based on the transcriptome and metabolome data, Pearson’s correlation tests were used to explore the correlations between the DEGs and differential accumulated metabolites (DAMs). Only the detected correlations with a Pearson’s correlation coefficient (PCC) value ≥ 0.9 and P ≤ 0.01 were selected. Cluster analysis of DAMs and DEGs was conducted on MetaboAnalyst 5.0 website (https://www.metaboanalyst.ca/) to further reveal the valve role of *SmDXS5* gene in regulating carbon flux of primary and secondary metabolism of *S. miltiorrhiza.* Based on the transcriptome gene expression and metabolite expression correlation analysis results, further screening of higher correlation analysis results using Cytoscape software (https://cytoscape.org/) to draw the gene-metabolite correlation network diagram.

## Results

### Culture of transgenic hairy roots, PCR analysis and copy number determination

Plasmids containing the cDNAs encoding *SmDXS5* driven by CaMV 35S promoter, were introduced into *S. miltiorrhiza* by using disarmed *A. tumefaciens* C58C1 strain. Hairy roots with normal phenotype and normal growth were selected for further analysis. The leaves of *S. miltiorrhiza* sterile seedling were co-cultured with *A. tumefaciens* for 2 days. Growth situation was shown in **Figure S1A**, and the leaves of explants were in good condition. After infection for 15 days, the *S. miltiorrhiza* explants grew hairy roots, as shown in **Figure S1B**. Primers containing the promoter CaMV sequence and reverse primers of the target gene were designed for PCR verification. *rolC* and *rolB* genes carried by *A. tumefaciens* were used to verify positive.

Genomic DNA of hairy root of *S. miltiorrhiza* was extracted and the N-terminal region of *SmDXS5* gene and CaMV 35S promoter were amplified by PCR with specially designed primers. *rolC* and *rolB* genes carried by *A. tumefaciens* C58C1 were used for positive verification. The genes and primers were shown in **Table S2**. The lowercase fragment was the homologous sequence of the vector, and the uppercase fragment was the homologous sequence of the target gene. The length of the PCR fragment was 3576 bp (**Figure S2A**), which was consistent with the length of the target fragment on the designed recombinant vector. The length of PCR amplified fragment was 626 bp and 423 bp respectively, which was consistent with the length of *rolC* and *rolB* genes (**Figure S2B**). The above PCR results indicated that the hairy roots of *S. miltiorrhiza* transgenic with *SmDXS5* gene were successfully obtained.

**Figure 1:**
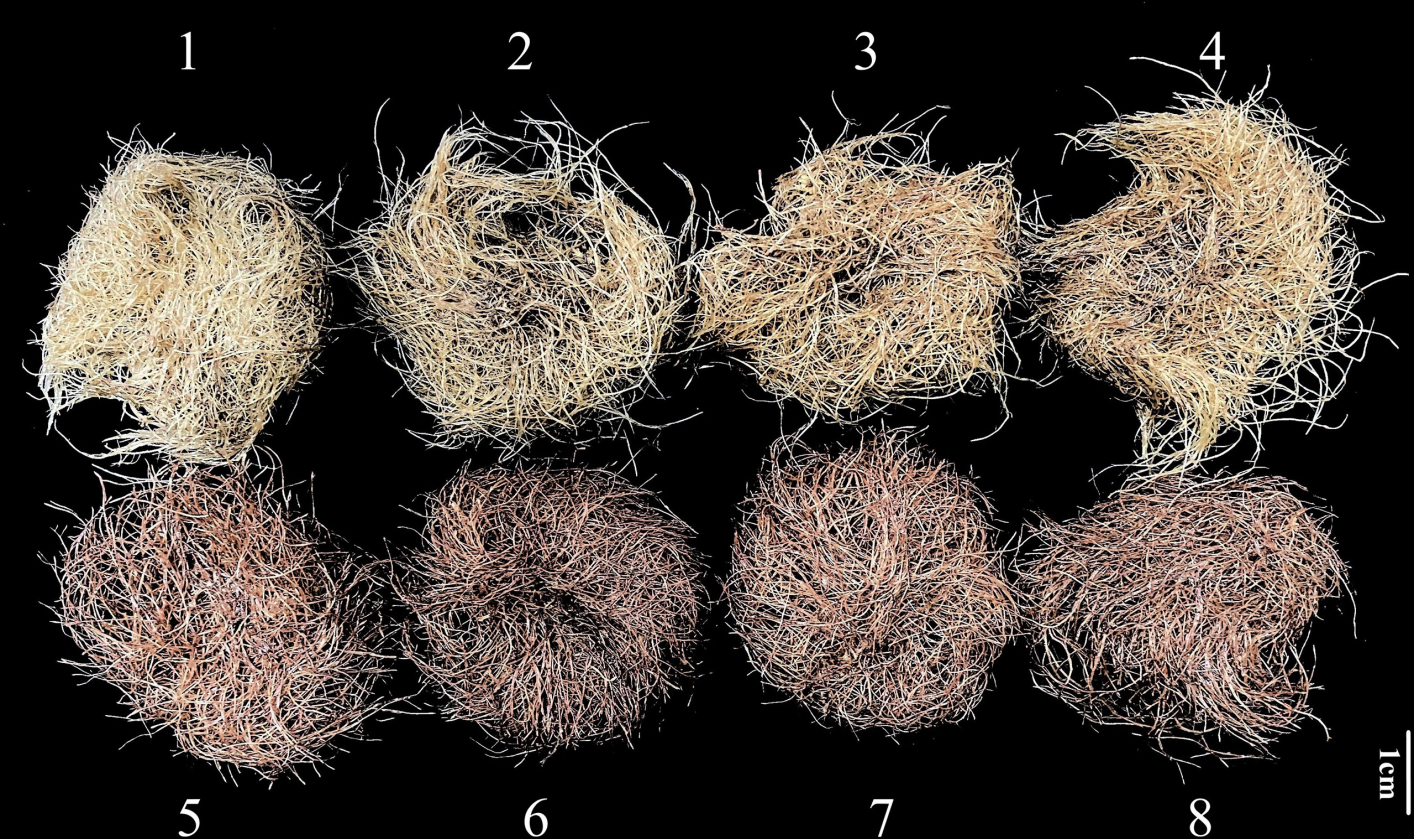
Culture of hairy roots of transgenic *S. miltiorrhiza*. **(1-4)** The blank control group. **(5-8)** The *SmDXS5* overexpression group.

Hairy roots cultured for 20 days (**Figure 1**) could be seen from the phenotype that *SmDXS5* overexpression group turned significantly red compared with the blank control (BC) group, this might be due to the tanshinone accumulation.

Primers D5-F and D5-R were used for qRT-PCR amplification of 8 standard samples, and each sample was repeated 5 times (**Table S3**). According to the data in the table, taking the standard copy number concentration as the abscissa and the measured Ct value as the ordinate, the calculated normalized logarithm equation was y = -1.451ln (x) + 34.434, R² = 0.985. The qRT-PCR primer fusion curve of *SmDXS5* gene showed a single peak (**Figure S3**), indicating that the primer specificity was good, and further sample qRT-PCR experiment could be carried out.

### Metabolomic profiling on hairy roots primary and secondary metabolism

**Figure 2:**
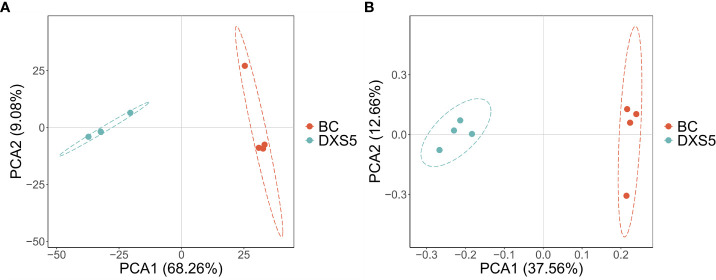
Principal component analysis (PCA) score plots. **(A)** Positive ion mode. **(B)** Negative ion mode.

After the samples were analyzed by LC-MS, the total ion flow in positive and negative ion modes was shown in **Figures S4A-D**. In this experiment, a total of 35453 peaks were detected in negative ion mode, and a total of 65535 peaks were detected in positive ion mode. PCA method was used to obtain generalized separation of all group variations. As shown in **Figures 2A, B**, all samples were distributed within 95% confidence interval. Positive ions (R2X=0.769, Q2=0.612) and negative ions (R2X=0.769, Q2=0.524) indicated that PCA model was accurate and reliable. PCA score diagram showed that *SmDXS5* overexpression group and BC group could be significantly separated under positive and negative ions, indicating that the metabolites of *SmDXS5* overexpression group and BC group had different changes and DAMs.

**Figure 3:**
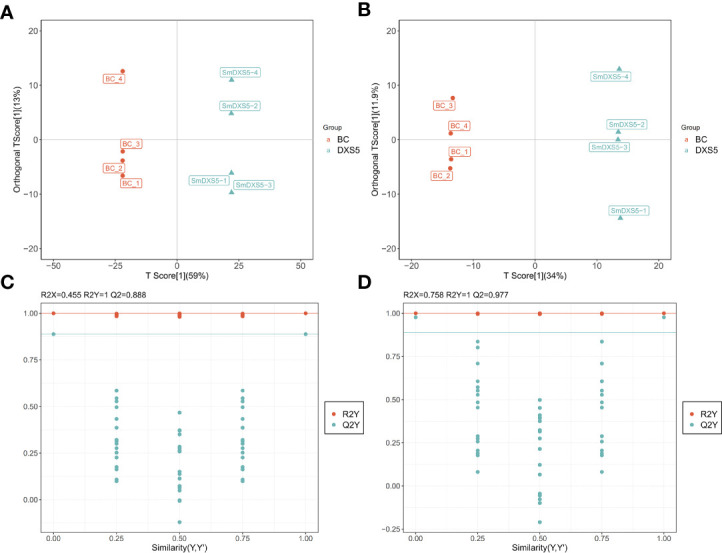
Orthogonal partial least squares-discriminant analysis (OPLS-DA) and 200-response sorting tests of the OPLS-DA model. **(A)**, **(C)** Positive ion mode. **(B)**, **(D)** Negative ion mode. The 200-response sorting tests of the OPLS-DA model, Q2 is an important parameter for evaluating the OPLS-DA model, and the R2X and R2Y represent the percentage of OPLS-DA model that can explain X and Y matrix information, respectively.

Orthogonal partial least squares-discriminant analysis (OPLS-DA) is a statistical method of supervised discriminant analysis, which can be used to further find the differential metabolites in each group. The OPLS-DA model of *SmDXS5* overexpression group and BC group was shown in **Figures 3A, B**, with positive ion mode (R2X=0.73, R2Y=0.998, Q2=0.965) and negative ion mode (R2X=0.658, R2Y=0.999, Q2=0.962). The 200-response sorting tests of the OPLS-DA model (**Figures 3C, D**), Q2 is an important parameter for evaluating the OPLS-DA model, and the R2X and R2Y represent the percentage of OPLS-DA model that can explain X and Y matrix information, respectively. And there was no over-fitting phenomenon, indicating that the established OPLS-DA model was effective and stable, and could well explore the DAMs.

**Figure 4:**
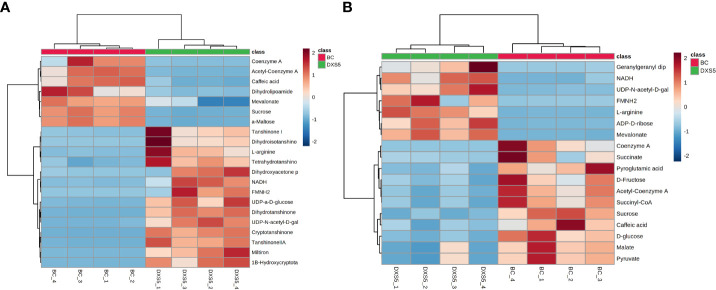
Cluster analysis heat map of differential metabolites. **(A)** Positive ion mode. **(B)** negative ion mode. In the above heat map, the red color indicates significant metabolites accumulation, and the blue color indicates a significant reduction in metabolites content.

In this experiment, the two groups were compared based on the OPLS-DA method, and the DAMs were further screened with absolute log 2 (fold change) ≥ 1, p-value < 0.05, and variable importance in projection (VIP) ≥ 1. Then, according to the mass-to-charge ratio (m/z) in the positive and negative ion mode, the human metabolome database and Metlin database (http://metlin.scripps.edu) were searched at the same time, taking the error of less than 10 ppm as the standard, the potential DAMs were finally inferred. According to the above method, there were 29 DAMs in positive ion mode and 24 DAMs in negative ion mode, as shown in **Table S4**. Compared with BC group, the up-regulated metabolites included arginine, dihydrotanshinone, cryptotanshinone, tanshinone II A, tetrahydrotanshinone, 1β-hydroxyl cryptotanshinone, dihydroisotanshinone I, tanshinone I, mainly secondary terpenoids. The down-regulated metabolites included sucrose, glucose, CoA, acetyl CoA, succinyl CoA, malic acid, succinic acid, pyruvate and other primary metabolites and caffeic acid. According to the DAMs identified, we use MetaboAnalysis 5.0 website analysis tool (https://www.metaboanalyst.ca/) to annotate the path. The DAMs were combined with sample clustering to draw a heat map. The colors represented the peak intensity of metabolites, with red representing high expression and blue representing low expression. As shown in **Figures 4A, B**, in positive and negative ion mode, BC group and *SmDXS5* overexpression group could be clustered into one group respectively, indicating differences in metabolites between the two groups. Caffeic acid, arginine, sucrose, glucose, CoA, acetyl CoA, succinyl CoA, malic acid, succinic acid and pyruvate were clustered with BC group, while secondary metabolites tanshinones and amino sugars were clustered with *SmDXS5* overexpression group. Cluster analysis showed that the carbon flux in *S. miltiorrhiza* was distributed in different metabolic pathways.

### RNA-seq and assembly and functional annotation

As can be seen from the agarose gel electrophoresis results, RNA bands of the 8 samples in the two groups were clear and bright without obvious contamination or degradation (**Figure S5**). Total RNA absorbance results and sequencing data (**Table S5**, **S6**) showed that the quality of a total of 8 samples in the BC group and the *SmDXS5* overexpression group all met the requirements and could be used for subsequent transcriptomic tests. Combined with the comparative analysis of the known reference genome of *S. miltiorrhiza* (**Table S7**), all gene sequences and information could be obtained more accurately. Genetic information could be obtained more comprehensively by analyzing six databases at the same time. The result of functional annotation of the six databases showed (**Figure S6**) that the number of genes annotated by different databases varies greatly, with NR database having the most and KEGG database having the least.

### Analysis of differentially expressed genes (DEGs)

A total of 36102 expressed genes were detected in this analysis, including 26752 known genes and 9350 new genes. There were 58534 expressed transcripts, including 25191 known transcripts and 33343 new transcripts. All differential transcripts of BC group and *SmDXS5* overexpression group were compared. Taking p-adjust < 0.05 and | log2fc | ≥ 1 as the screening criteria, the results showed that there were 18646 DEGs. Compared with BC group, 8994 genes were up-regulated and 9652 genes were down-regulated.

**Figure 5:**
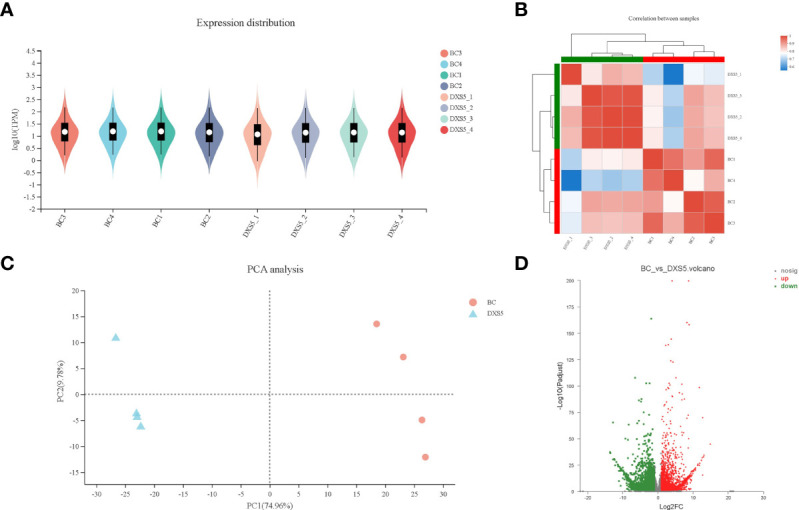
Analysis of differentially expressed genes (DEGs). **(A)** differentially expressed genes expression distribution. **(B)** differentially expressed genes correlation analysis between samples. **(C)** principal component analysis (PCA) of each group. **(D)** Volcano plot of differentially expressed genes between the 2 libraries, red and green dots represent the significantly upregulated and downregulated genes.

The gene expression levels of all samples were similar (**Figure 5A**). PCA analysis could show the separation trend of the transcriptome between the two groups of samples, indicating whether there was a difference in the transcriptome between the two groups (**Figures 5B, C**). The results showed that there was a large difference between the two groups of samples, and the transcriptome had a good separation trend. In order to display the overall changes of DEGs more directly, the screened DEGs were plotted into volcano maps to analyze the up-regulation changes of genes (**Figure 5D**).

### DEGs identification and enrichment analyses

**Figure 6:**
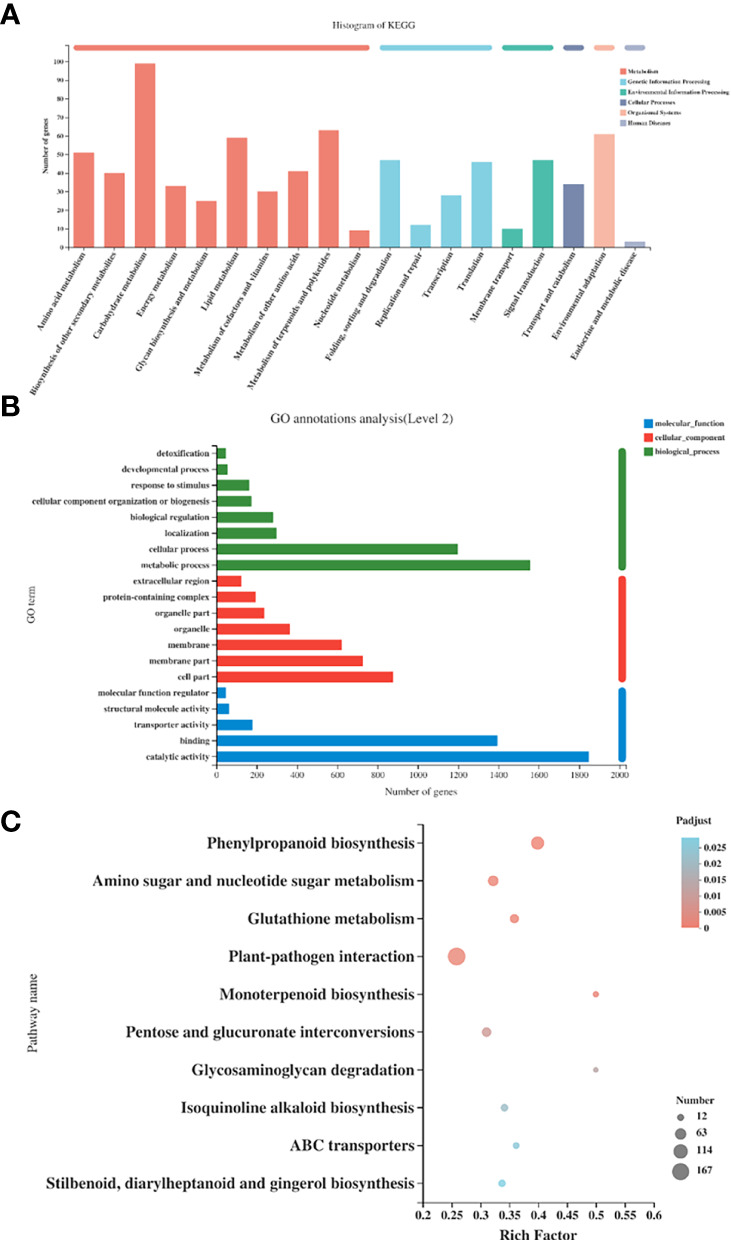
Functional GO and Kyoto Encyclopedia of Genes and Genomes (KEGG) pathway classification of differentially expressed genes. **(A)** KEGG annotation histogram of differentially expressed genes. **(B)** GO annotation histogram of differentially expressed genes. **(C)** Top 20 KEGG pathways with the most significant enrichment in the 2 groups.

The RPKM values were calculated for each unigene by setting |log2(fold change)| ≥ 2 and P ≤ 0.05 as thresholds for significant DEGs selection. We performed KEGG functional annotation for all DEGs in the two groups (*SmDXS5* overexpression group and BC group). As shown in **Figure 6A**, it was found that DEGs were mainly annotated into two categories of metabolism and genetic information processing. Among them, many subcategories were designed, such as “carbohydrate metabolism”, “metabolism of terpenoids and polyketides”, “lipid metabolism”, “environmental adaptation”, “signal transduction”, “folding, sorting and degradation”, etc.

GO function annotation was performed for all DEGs in *SmDXS5* overexpression group and BC group (**Figure 6B**). The results showed that compared with the BC group, *SmDXS5* overexpression group DEGs was mainly annotated into 20 functional subcategories. There were many categories involved, including “metabolic process”, “cellular process”, “catalytic activity”, “binding”, “membrane component” and “membrane”.

In order to further analyze the biological processes involved in DEGs between *SmDXS5* overexpression group and BC group, KEGG was used for functional enrichment analysis, and a total of 129 metabolic pathways were identified. P-value ≤ 0.05 was considered as significant enrichment, and a total of 10 metabolic pathways were significantly enriched (**Figure 6C**).

GO enrichment analysis was conducted for all identified differential genes, and p-value ≤ 0.05 was considered as significant enrichment. Among them, the main enrichment subcategories of biological processes include: metabolic process, oxidation-reduction process, carbohydrate metabolism process, drug metabolic process. The main enrichment subcategories of cellular component include membrane, extracellular region, and cell wall. The main rich subcategories of molecular functions include: catalytic activity, oxidoreductase activity, cofactor binding, etc. (**Table S8**).

### Analysis of DEGs in primary and secondary metabolic pathways

According to KEGG and GO annotation results, 19 genes related to terpenoid synthesis, 17 genes related to glucose metabolism, 11 genes related to TCA cycle, 7 phenylpropane biosynthesis genes, and 32 UDP-glycotransferases were identified according to KEGG pathway enrichment and GO functional annotation results, as shown in **Table S9**. Data analysis showed that the DEGs in terpenoid pathway were significantly up-regulated, and the genes in phenylpropane pathway were significantly down-regulated, which was consistent with the changes of secondary metabolites. Secondly, in primary metabolism, as a glycolysis pathway providing precursor carbon source, this pathway was observed to be more active and its metabolic enzyme gene expression was upregulated. At the same time, most genes in TCA cycle and pyruvate metabolism were up-regulated. The results showed that overexpression of *DX5* gene had a positive regulation effect on genes related to primary metabolism and secondary terpenoid synthesis of hairy roots of *S. miltiorrhiza*, including *DXS*, *DXR*, *MDS*, *HDS*, *HDR*, *AACT*, *HMGR*, *HMGS*, *MDC*, *GPPS*, *CPS*, *KSL* and *CYP76AH1*. In addition, it negatively regulates the synthesis of secondary phenylpropanoid pathways, including *PAL*, *C4H*, *TAT*, *4CL*, etc. Meanwhile, the expression levels of *MYB36* transcription factor and annotated 25 UDP-glycosyltransferase genes related to transcription regulation were also significantly increased.

**Figure 7:**
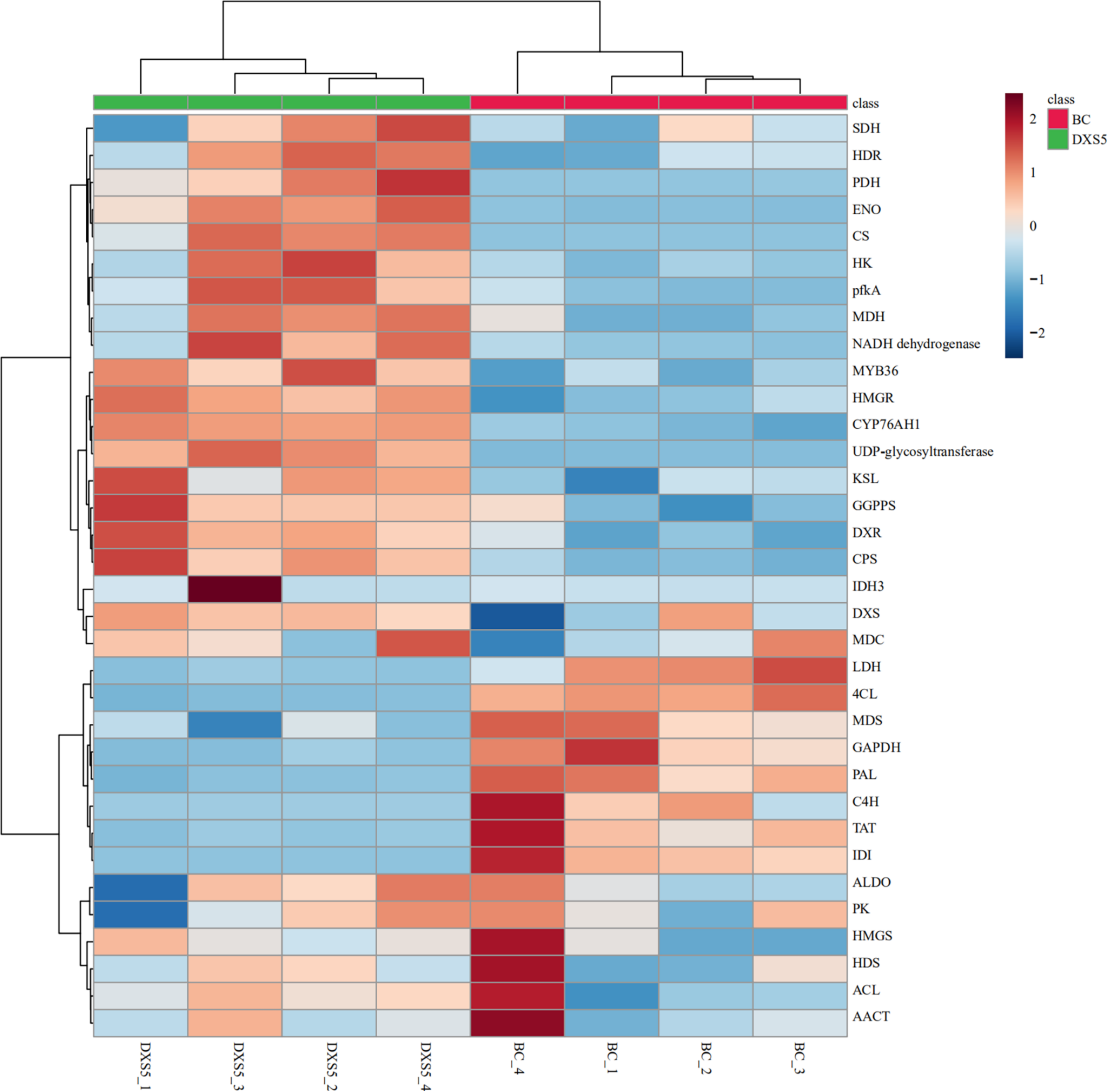
Cluster analysis of differentially expressed genes (DEGs).

DEGs in major pathways were combined with samples for cluster analysis using MetaboAnalyst 5.0 website (https://www.metaboanalyst.ca/). The results showed that *SmDXS5* overexpression group and NC group could be clustered into one group in DEGs, indicating differences between the two groups. Most of the genes involved in glycolysis, TCA cycle and terpenoid synthesis were significantly positively correlated in *SmDXS5* overexpression group, while the enzymes involved in phenylpropane synthesis pathway and lactate dehydrogenase genes were significantly negatively correlated in *SmDXS5* overexpression group (**Figure 7**). Cluster analysis showed that *SmDXS5* overexpression had differential regulation on primary and secondary metabolic pathways of *S. miltiorrhiza*.

**Figure 8:**
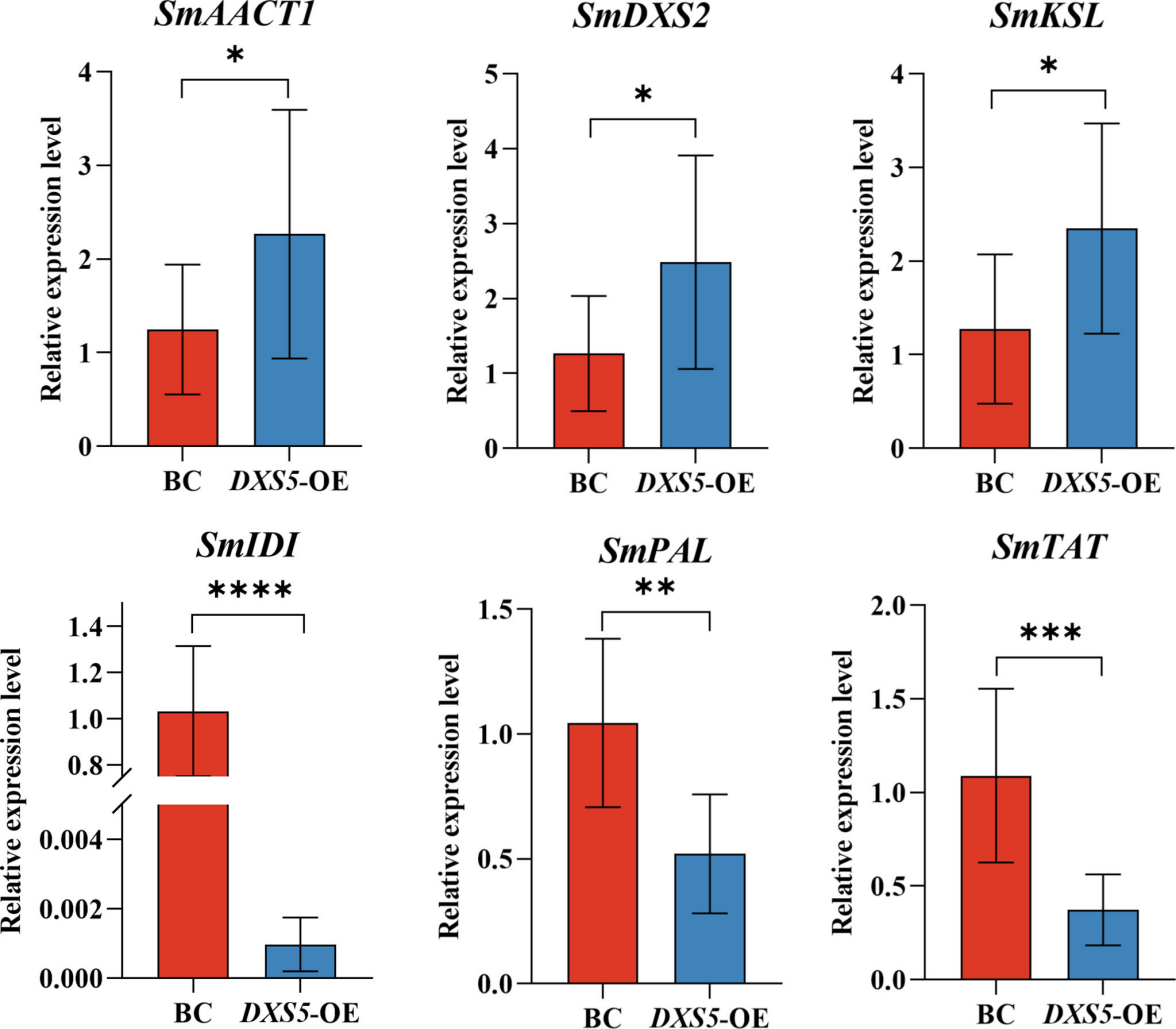
Relative expression levels of key enzyme ge*nes (SmAACT1, SmDXS2, SmKSL, SmIDI, SmPAL, SmTAT)*. *means p value < 0.05, **means p value < 0.01, ***means p < 0.001.

β-actin of hairy root of *S. miltiorrhiza* was used as reference gene, the relative expression levels of *SmAACT1*, *SmDXS2*, *SmKSL*, *SmIDI*, *SmPAL* and *SmTAT* genes in the hairy roots of *S. miltiorrhiza* were analyzed by 2^-ΔΔCT^ method (melting curves were shown in **Figure S7**, relative expression of the 6 genes based on transcriptome data were shown in **Figure S8**, **Table S10**). The results showed that the expression levels of the starting genes of terpenoid pathway *SmAACT1*, *SmDXS2* and *SmKSL* were higher than those of the BC group, while the expression levels of the starting genes of phenylpropanoid pathway *SmPAL* and *SmTAT* were lower than those of the BC group (**Figure 8**). The results data come from RT-PCR analysis, showed that the expression patterns of key rate-limiting enzyme genes were consistent with the transcriptome results.

### Correlation analysis between transcriptome and metabolome data

**Figure 9:**
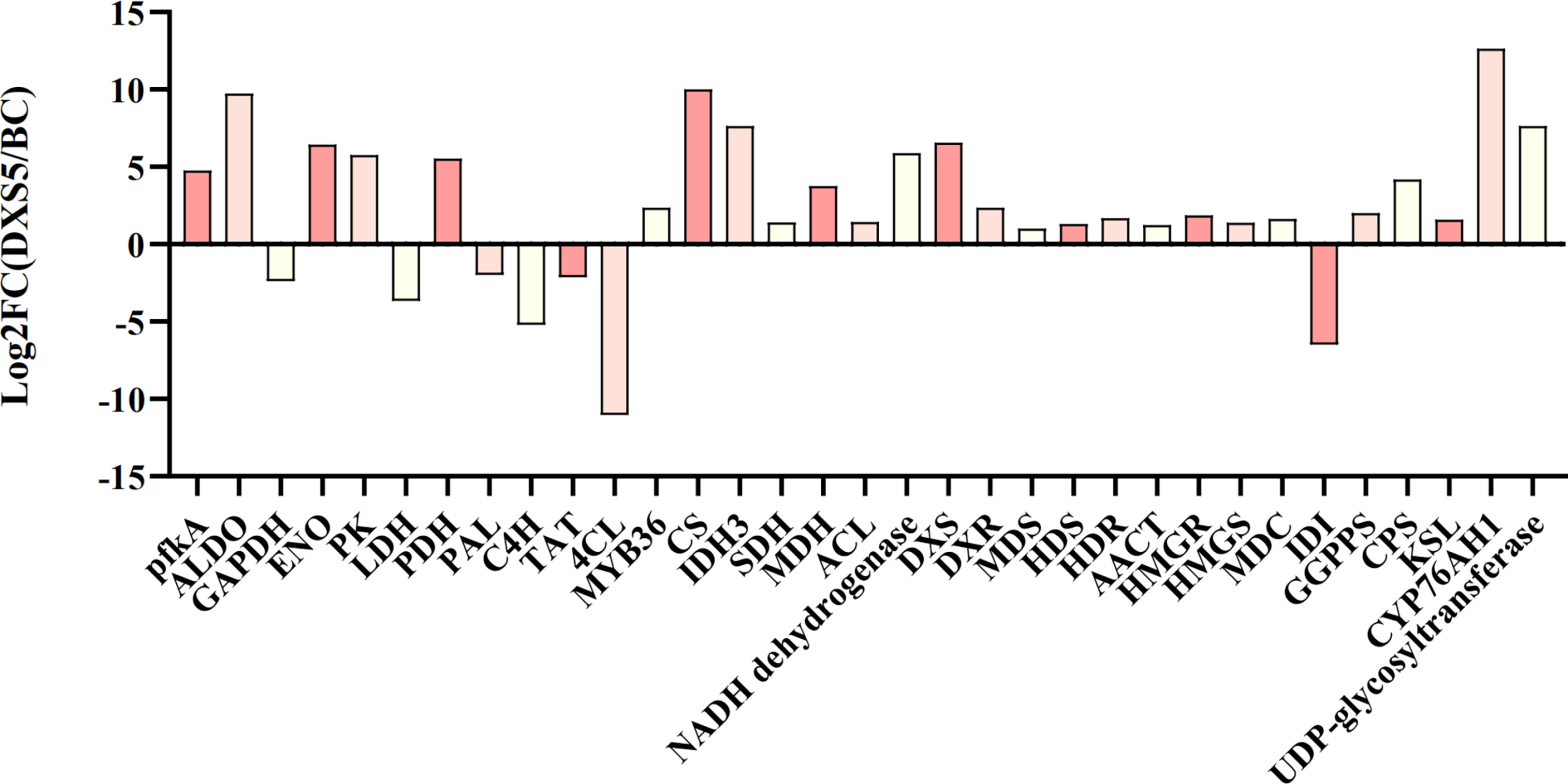
Log2FC value distribution of differentially expressed genes (DEGs).

This part is mainly based on the idea of data integration on the same KEGG annotation pathway, combining the basis of important pathways and metabolite changes, and discussing the relationship between them. According to its Log2FC (DXS5/BC) value, the histogram (**Figure 9**) showed that the genes of glycolysis, TCA cycle and terpenoid synthesis pathway were significantly up-regulated in *SmDXS5* group, while phenylpropane synthesis pathway and lactate dehydrogenase genes were down-regulated compared with BC group. These results suggest that *SmDXS5* overexpressed gene may regulate different pathways.

**Figure 10:**
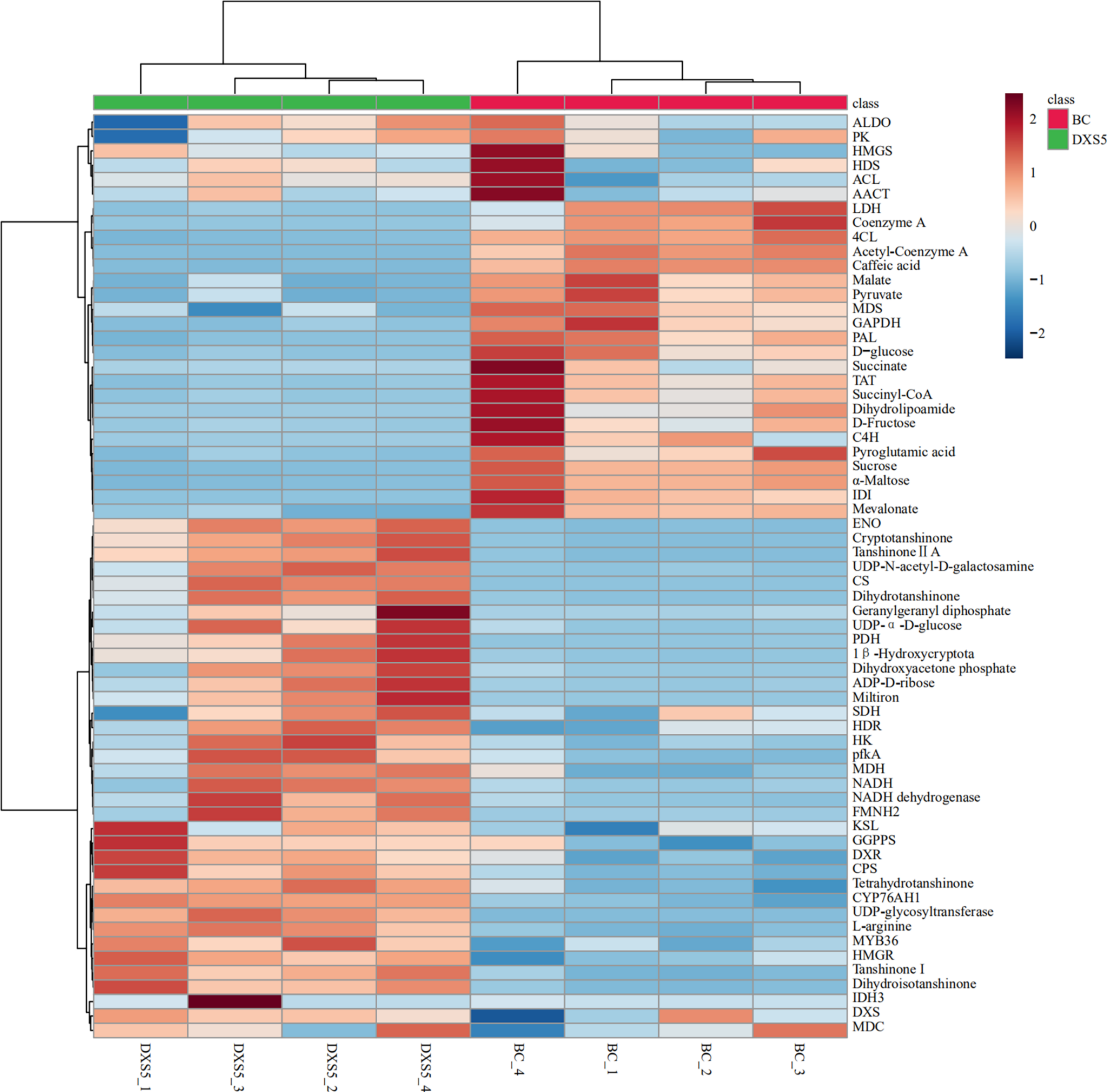
Heatmap based on hierarchical clustering analysis.

**Figure 11:**
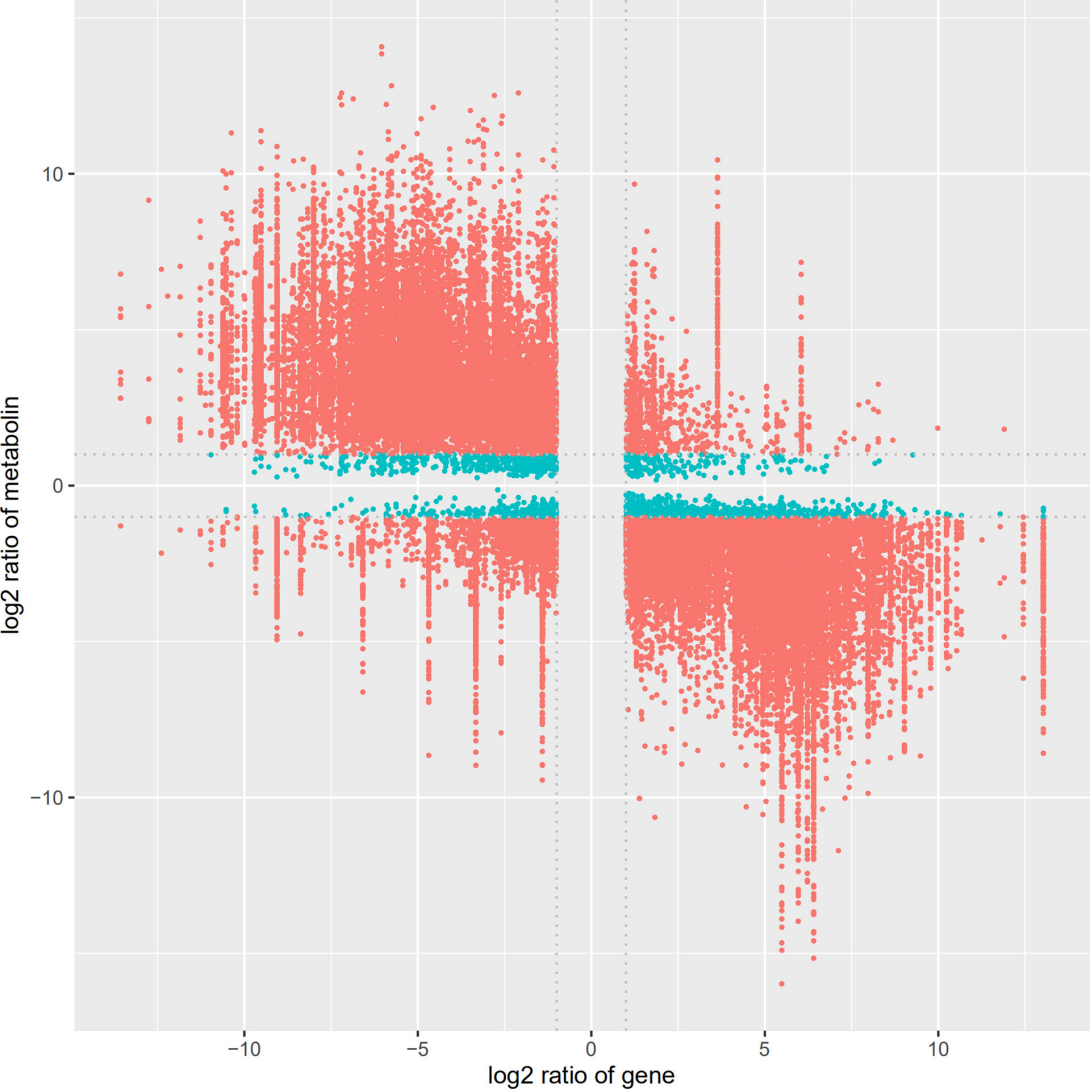
Nine quadrant diagram showing the correlation of differentially expressed genes and differential accumulated metabolites.

The identified differential metabolites and differentially expressed genes were combined, and cluster analysis was conducted using MetaboAnalyst 5.0 website, as shown in **Figure 10**. We can visually view the distribution of gene regulation and metabolite accumulation and find that there is a significant correlation between metabolites and genes. Among them, glycolysis genes and transcription factors involved in primary metabolism could be clustered together with terpenoid metabolite accumulation, which had a significant positive correlation with *SmDXS5* group. At the same time, the expression of sucrose, glucose, TCA cycle intermediates, caffeic acid and phenylpropane biosynthesis genes could be clustered together, and had a significant positive correlation with BC group. Cluster analysis results further intuitively revealed that *SmDXS5* overexpressed gene can regulate metabolic carbon fluxes in different metabolic pathways through molecular regulation, demonstrating the role of *SmDXS5* valve gene. Based on gene expression and transcriptome metabolites to express the correlation analysis results, respectively, based on gene - metabolites (i.e., each gene corresponding to the correlation of the highest metabolites) selected corresponding relation, metabolites, gene (i.e., the correlation of each metabolite selected corresponding to the highest gene) corresponding relationship, combined with gene and metabolites logFC information difference between the groups, nine quadrant diagram was drew (**Figure 11**).

**Figure 12:**
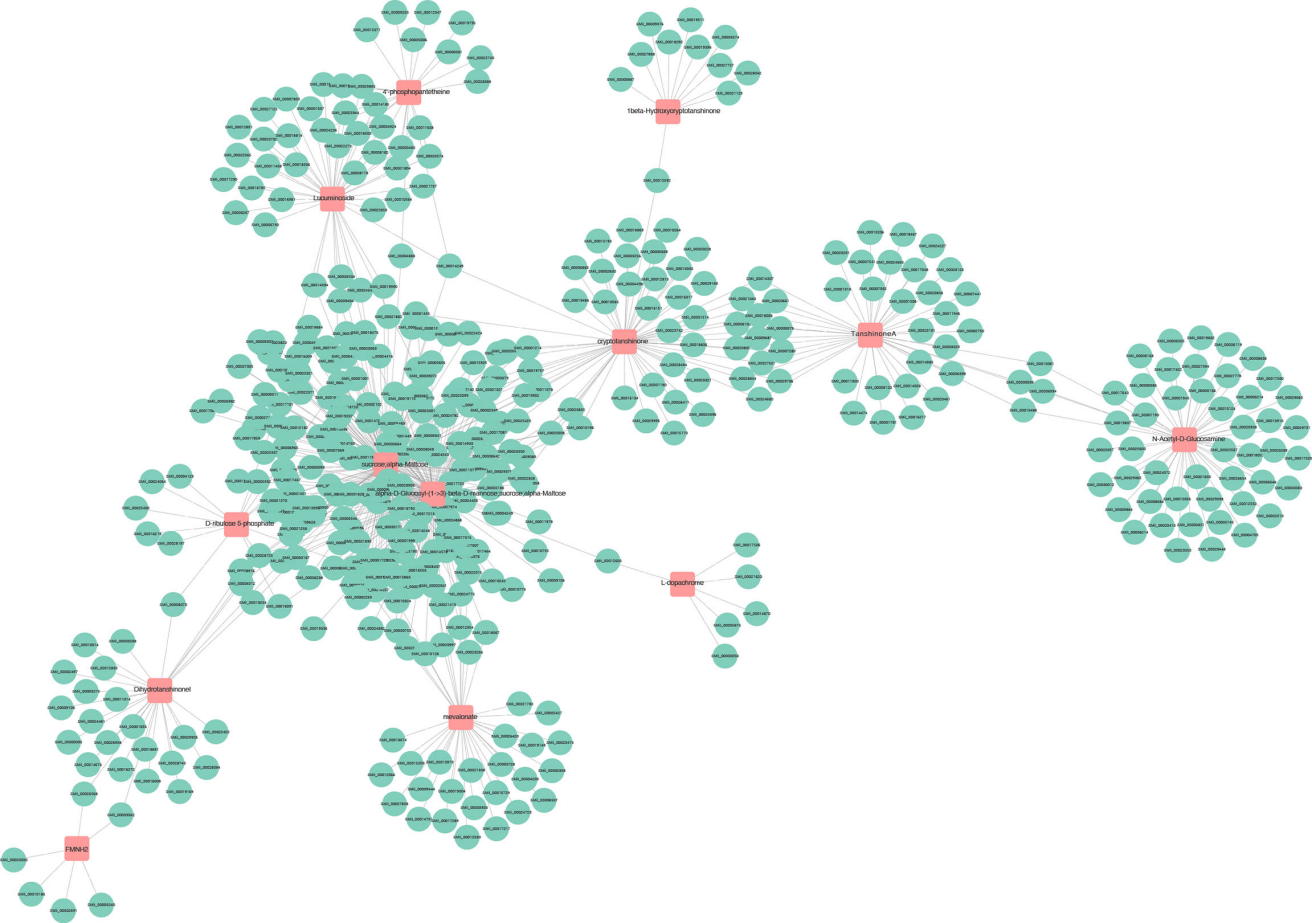
Connection network between differentially expressed genes (green circles) and differential accumulated metabolites (red squares).

The transcriptome and metabolome data were also compared by a Pearson correlation analysis. Among them, a single gene was regulated by multiple metabolites, or a single metabolite was regulated by multiple genes that are ubiquitous (**Figure 12**). Whether the line is connected indicates whether the correlation meets the threshold. The network with a total connection number less than 10 was excluded.

## Discussion

In this study, using the advantages of high-throughput screening of functional genes in the transcriptome and the characteristics of rapid multi-component analysis of the metabolome, it was concluded that overexpression of valve gene *SmDXS5* could significantly affect the transcription level of primary and secondary metabolic genes and the accumulation of metabolites.

### Terpenoid biosynthesis

Terpenes use isoprene as their molecular framework (C5 unit), and the synthesis of their C5 framework precursors IPP and DMAPP is carried out through MVA and MEP pathways, respectively (Lange et al., 2000). In this study, 5 DEGs, *SmAACT1*, *SmAACT2*, *SmHMGR1*, *SmHMGS* and *SmMDC*, annotated on MVA pathway, were significantly up-regulated when *SmDXS5* overexpression in hairy roots. *SmAACT* catalyzes the condensation of 2 primary metabolites acetyl CoA into acetyl CoA, initiating the first step reaction of MVA pathway (Wang et al., 2010). Transcriptional data of our experiment showed that the expressions of *SmDXS1*, *SmDXS2*, *SmDXS3*, *SmDXR*, *SmMDS*, *SmHDS* and *SmHDR1* annotated by MEP pathway were significantly up-regulated. IPP and DMAPP can be converted into each other under the catalysis of IDI enzyme (Nagegowda, 2010). Different amounts of IPP and DMAPP can combine to form monoterpene, sesquiterpene, diterpene, triterpene and tetraterpene precursors under the catalysis of different isopentene transferases (McGarvey and Croteau, 1995; Liang et al., 2002), and these isopentene transferases (GPPS, FPPS, GGPPS) are significantly upregulated in the transcriptome. In the downstream pathway of tanshinone synthesis using GGPP as precursor, the required *SmCPS* and *SmKSL* genes were significantly increased compared with the BC group (Gao et al., 2009; Ma et al., 2012; Guo et al., 2013). These results were consistent with the metabolome results, and the downstream tanshinones were significantly increased, suggesting that *SmDXS5* has a positive regulation effect on key terpenoids synthesis pathway under the regulation of overexpression *SmDXS5* gene.

Cytochrome P450 (CYP450) is a kind of self-oxidizing heme multifunctional protein family, which can catalyze a variety of oxidation reactions and play an important regulatory role in plant growth and secondary metabolite accumulation (Manikandan and Nagini, 2018). In the downstream pathway of terpenoid synthesis, *CYP76AH1* can catalyze miltiradiene to synthesize ferruginol (Mao et al., 2020), which is a key rate-limiting enzyme in tanshinone synthesis. In this study, the transcriptome data were analyzed and 304 differentially expressed CYP450 genes were annotated. The expression of *CYP76AH1* gene was significantly increased in *SmDXS5* group, indicating that the expression of this gene promoted the accumulation of tanshinones in the downstream pathway.

### Phenylpropane biosynthesis

The main phenolic acids in the secondary metabolites of *S. miltiorrhiza* were rosmarinic acid (RA) and salvianolic acid B, both of which could be considered as caffeic acid derivatives, and RA might be used as a precursor compound for complex salvianolic acid compounds (Petersen and Simmonds, 2003). The secondary biosynthesis of RA is mainly carried out through two parallel pathways, phenylalanine and tyrosine-drived. Phenylalanine ammonia-lyase (PAL) is a rate-limiting enzyme in the first step of phenylpropane metabolic pathway, which catalyzes the synthesis of cinnamic acid from L-phenylalanine (Yun et al., 2015). The first step reaction on another tyrosine pathway is catalyzed by tyrosine aminotransferase (TAT) and the product of this pathway is 4-hydroxyphenyllactic acid (4-HPLA), which is also a precursor metabolite of salvianic acid. The products of these two pathways provide precursors for the synthesis of downstream RA. In this experiment, *SmPAL2*, *SmPAL3*, *SmTAT*, *SmC4H* and *Sm4CL2* genes annotated by transcriptome were significantly down-regulated in *SmDXS5* overexpression group, which was consistent with the results of metabolic composition determination. These results indicated that *SmDXS5* overexpressed hairy roots inhibited the synthesis of phenolic acids by negatively regulating the expression of enzymes in the phenylpropane pathway.

Changes in metabolic flow are caused by changes in the expression of genes in metabolic pathways. Transcription factors can regulate changes in primary metabolic flow by activating or inhibiting the expression of key enzyme genes. MYB transcription factor is generally involved in the regulation of primary and secondary metabolism and abiotic and biological stress in plants (Dubos et al., 2010; Liu et al., 2015). Studies have found that overexpression of *SmMYB36* transcription factor can promote tanshinone accumulation in hairy roots of *S. miltiorrhiza*, but inhibit the biosynthesis of phenolic acids and flavonoids (Ding et al., 2017). Transcriptome annotation in this experiment showed that the expression of 3 *SmMYB36* genes increased in *SmDXS5* overexpression group, which was speculated to be the main reason for the decrease of salvianolic acid content.

### Glycolysis

The hairy roots of *S. miltiorrhiza* cultured in dark environment cannot carry out photosynthesis, so their carbon source comes from sucrose in the medium. Glycolysis is an important metabolic pathway widely existing in prokaryotic and eukaryotic organisms, mainly in the cytoplasm, through which sugars are catalyzed by a series of enzymatic reactions, which not only provide energy for hairy root growth and development, but also ultimately convert monosaccharides into pyruvate as a carbon source for other biosynthesis (Plaxton, 1996; Fernie et al., 2004). In this experiment, the content of glucose, sucrose and maltose in hairy roots of *SmDXS5* overexpression group decreased, while the content of dihydroxyacetone phosphate (DHAP), the intermediate product of glycolysis, was significantly accumulated. The expression levels of hexokinase (HK), 6-phosphofructokinase (pfkA), glyceraldehyde-3-phosphate dehydrogenase (GAPDH), enolase (ENO) and pyruvate kinase (PK) were significantly increased. These results indicated that under the regulation of *SmDXS5* overexpression, glycolysis pathway was activated, and sugars were utilized to synthesize downstream carbon sources. However, metabolome data showed that in *SmDXS5* hairy roots, the contents of pyruvate and acetyl CoA were decreased when glycolysis was activated, which we speculated was used by secondary biosynthesis. Pyruvate can be converted to lactic acid in the last step of glycolysis, and the reaction is catalyzed by L-lactate dehydrogenase (LDH). At the same time, it can also be catalyzed by pyruvate dehydrogenase complex (PDHC) on the inner membrane of mitochondria to generate acetyl CoA (Li et al., 2019), which can enter the tricarboxylic acid cycle as an intermediate product. It can also enter the MVA pathway as a starting substrate for terpenoid synthesis. Combined with transcriptome data, the expression of *SmLDH* decreased and the expression of 5 annotated *SmPDH* subunits increased.

## Conclusions

We overexpressed *SmDXS5* as a ‘valve’ gene regulating the intersection of diterpenoid secondary and primary metabolic pathways. Combined with the analysis of transcriptome-related genes and metabolome-related DAMs, this regulation increased the contents of diterpenoids and the expression of synthetic genes. It also inhibits the accumulation and synthesis of phenolic acids. Thus, the key role of *SmDXS5* in the regulation of primary and secondary metabolic flow balance of terpenoids has been identified. However, the interaction between *SmDXS5* and its upstream and downstream key proteins is not discussed here in depth, and the specific mechanism of regulation was not analyzed. This is a deficiency of our paper. However, our study has positive significance for improving the quality of *S. miltiorrhiza* and other medicinal plants and for increasing the content of medicinal active ingredients in TCM. The discovery of this ‘valve’ gene function of *SmDXS5* provides a basis for further research on the regulatory mechanism of target genes, biosynthesis pathways and directional cultivation of *S. miltiorrhiza*.

## Data availability statement

The datasets presented in this study can be found in online repositories. The names of the repository/repositories and accession number(s) can be found below: https://www.ncbi.nlm.nih.gov/genbank/, PRJNA851849.

## Author contributions

X-YW conceived the study and performed the experimental measurements, processed the experimental data. D-CZ, L-LL, and Z-RW integrated the data and wrote the manuscript. W-JX and J-LL performed the experimental measurements and helped in sampling. S-TT and J-HW performed the RNA extraction and the qRT-PCR experiments. YL, CZ, and CL analyzed the results and prepared the figures and tables. Zhirong Wang has made important contributions to data analysis and writing, Da-chuan Zhang and Zhirong Wang contribute equally to the article and are co-first authors of the article. All authors contributed to the article and approved the submitted version.

## Funding

This work was supported by the National Science and Technology Fundamental Resources Investigation Program of China (No. 2018FY100700), and the Science and Technology Support Plan of Guizhou Province (No. 20204Y074).

## Acknowledgments

The authors are thankful to the Beijing University of Chinese Medicine for the assistance in conducting this study.

## Conflict of interest

The authors declare that the research was conducted in the absence of any commercial or financial relationships that could be construed as a potential conflict of interest.

## Publisher’s note

All claims expressed in this article are solely those of the authors and do not necessarily represent those of their affiliated organizations, or those of the publisher, the editors and the reviewers. Any product that may be evaluated in this article, or claim that may be made by its manufacturer, is not guaranteed or endorsed by the publisher.

## Supplementary material

Supplementary Figure 1 The leaves of *S. miltiorrhiza* sterile seedling were co- cultured with *A. tumefaciens.*

Supplementary Figure 2 Detection by agarose gel electrophoresis.

Supplementary Figure 3 Melting curve of *SmDXS5.*

Supplementary Figure 4 Total Ion Chromatography of *SmDXS5* overexpression hairy roots group and BC group.

Supplementary Figure 5 Agarose gel electrophoresis results.

Supplementary Figure 6 Functional annotation of the six databases.

Supplementary Figure 7 qPCR melting curves of 6 genes.

Supplementary Figure 8 Heat map of relative expression of key enzyme genes of terpenoids and phenolic acids based on transcriptome data.

Supplementary Table 1 Transcript-specific primers and gene information.

Supplementary Table 2 Genes and primers.

Supplementary Table 3 qRT-PCR results of plasmid standard.

Supplementary Table 4 DAMs in positive ion mode and negative ion mode.

Supplementary Table 5 Total RNA absorbance results.

Supplementary Table 6 RNA-seq data.

Supplementary Table 7 Statistical table of comparison with reference genome.

Supplementary Table 8 GO annotation analysis of DEGs.

Supplementary Table 9 Differentially expressed gene list (SmDXS5 group and BC group).

Supplementary Table 10 Relative expression of key enzyme genes of terpenoids and phenolic acids based on transcriptome data.
