# Supplementary figures and images for: SmDXS5, acting as a molecular valve, plays a key regulatory role in the primary and secondary metabolism of tanshinones in Salvia miltiorrhiza
Source: Front Plant Sci. 2022 Nov 10;13:1043761. doi: 10.3389/fpls.2022.1043761 (PMC9685628; doi:10.3389/fpls.2022.1043761)

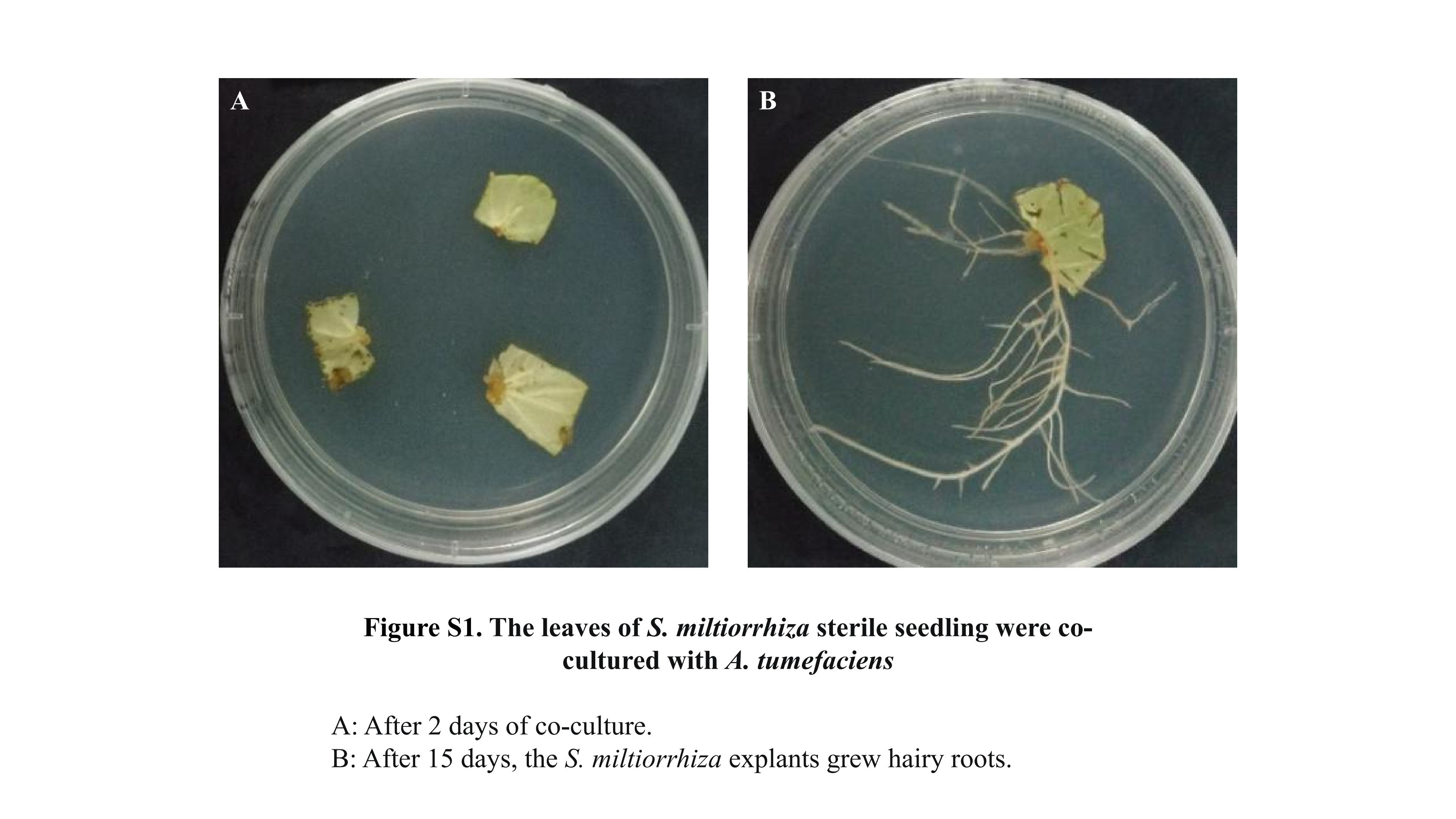

Supplement: Supplementary Figure 1 — The leaves of S. miltiorrhiza sterile seedling were co- cultured with A. tumefaciens. [file Image_1.png]

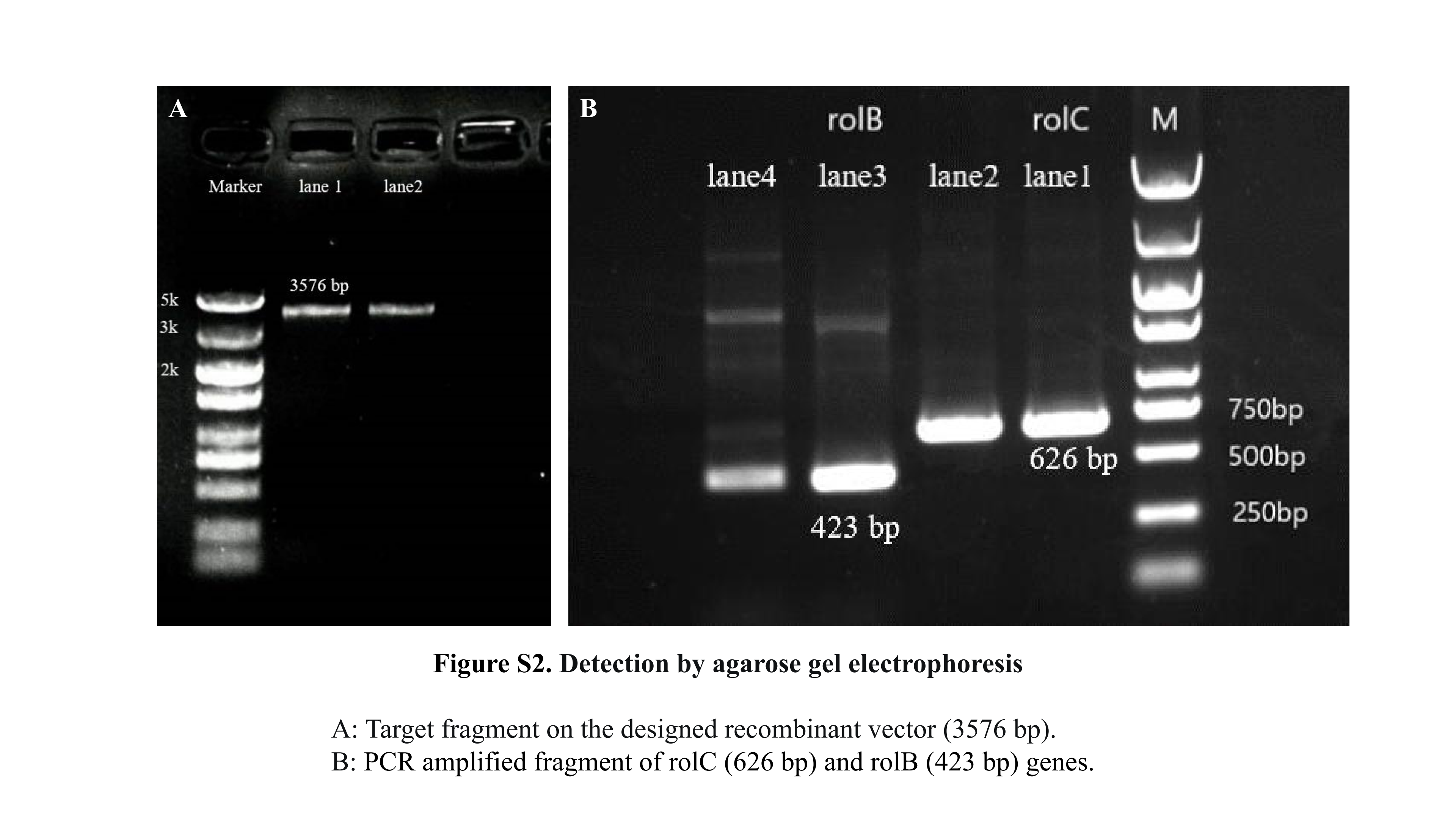

Supplement: Supplementary Figure 2 — Detection by agarose gel electrophoresis. [file Image_2.png]

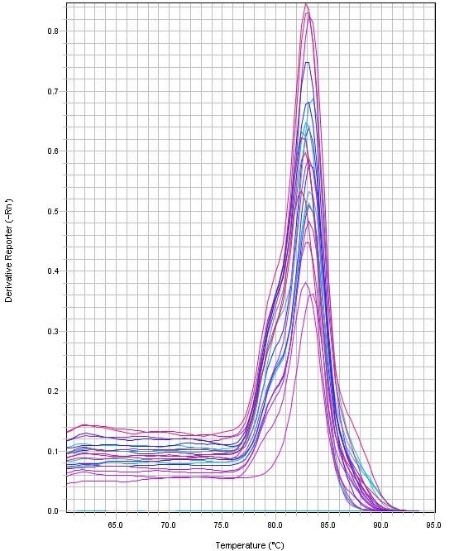

Supplement: Supplementary Figure 3 — Melting curve of SmDXS5. [file Image_3.jpeg]

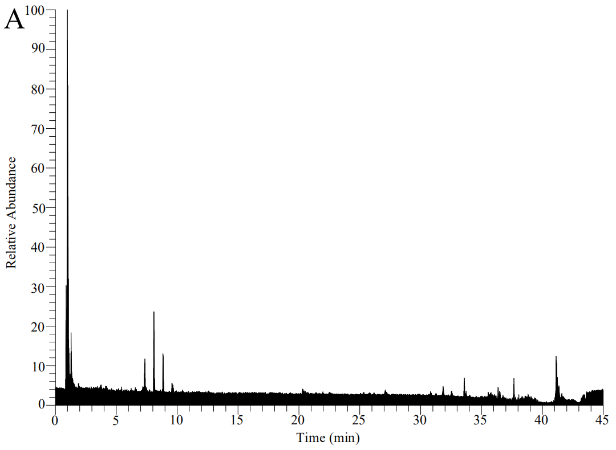

Supplement: Supplementary Figure 4 — Total Ion Chromatography of SmDXS5 overexpression hairy roots group and BC group. [file Image_4.jpeg]

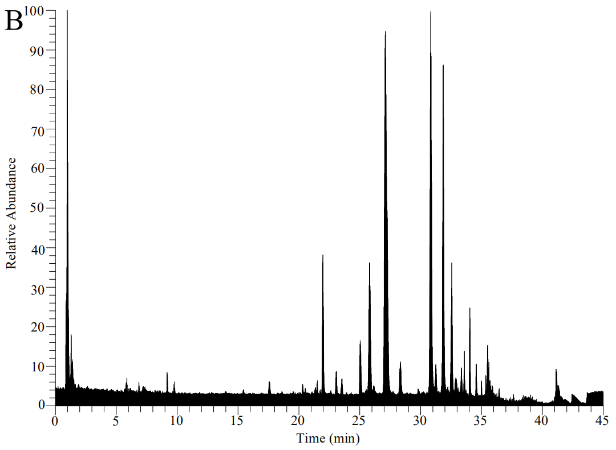

Supplement: Supplementary Figure 5 — Agarose gel electrophoresis results. [file Image_5.jpeg]

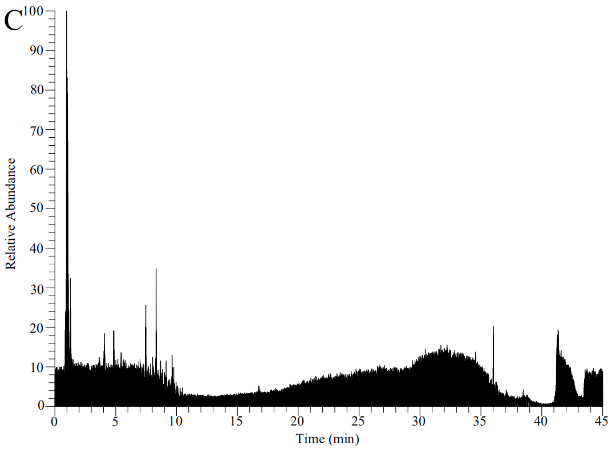

Supplement: Supplementary Figure 6 — Functional annotation of the six databases. [file Image_6.jpeg]

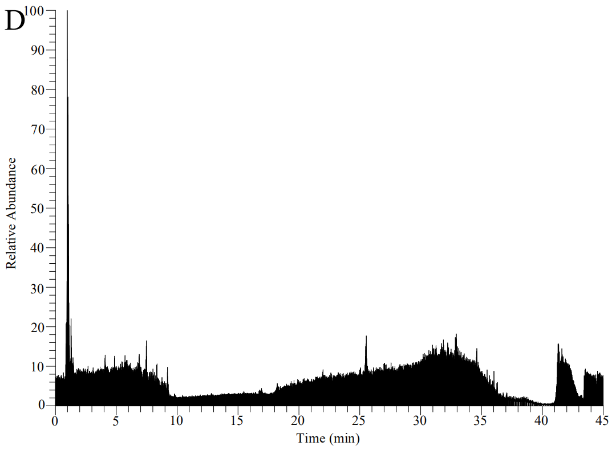

Supplement: Supplementary Figure 7 — qPCR melting curves of 6 genes. [file Image_7.jpeg]

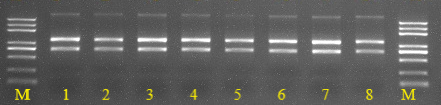

Supplement: Supplementary Figure 8 — Heat map of relative expression of key enzyme genes of terpenoids and phenolic acids based on transcriptome data. [file Image_8.jpeg]

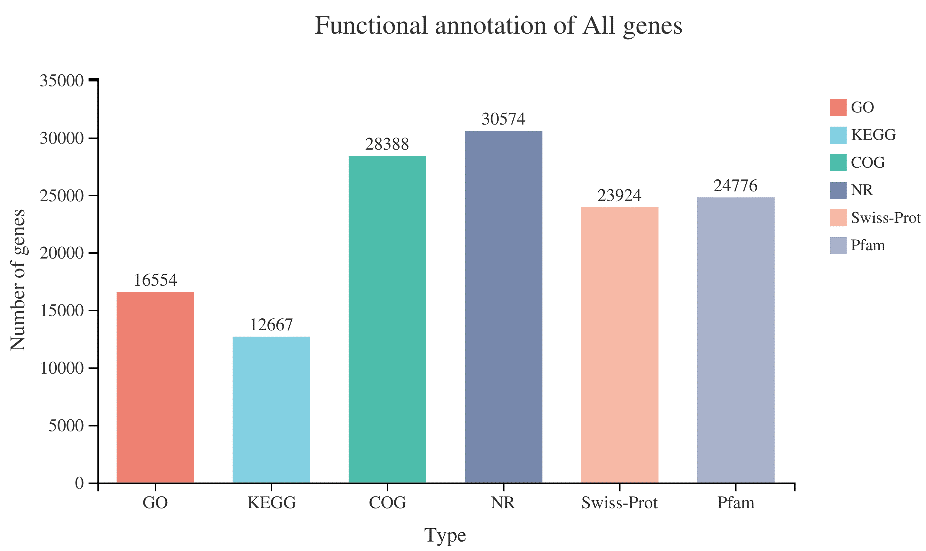

Supplement: Supplementary file 19 [file Image_9.jpeg]

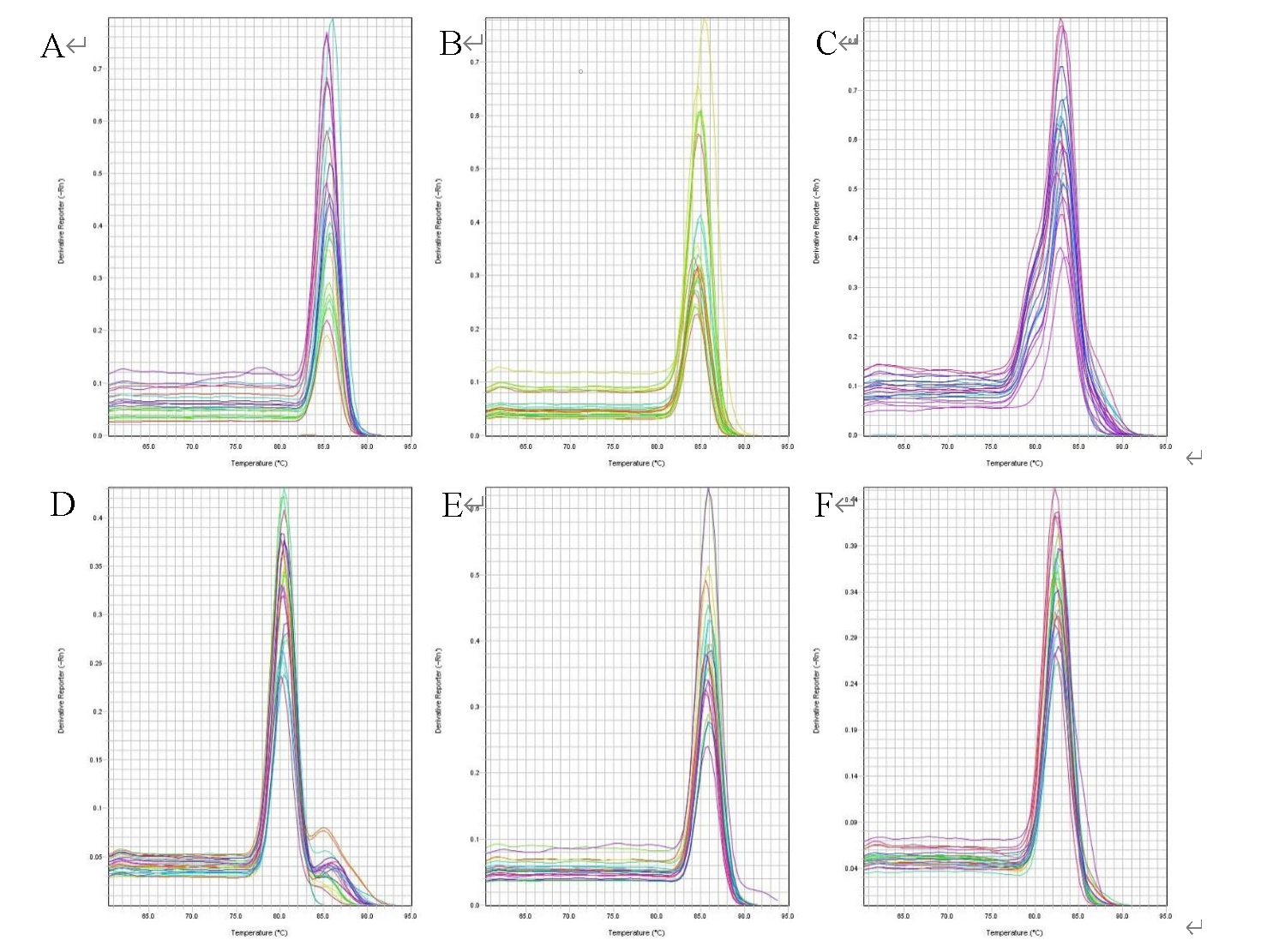

Supplement: Supplementary file 20 [file Image_10.png]

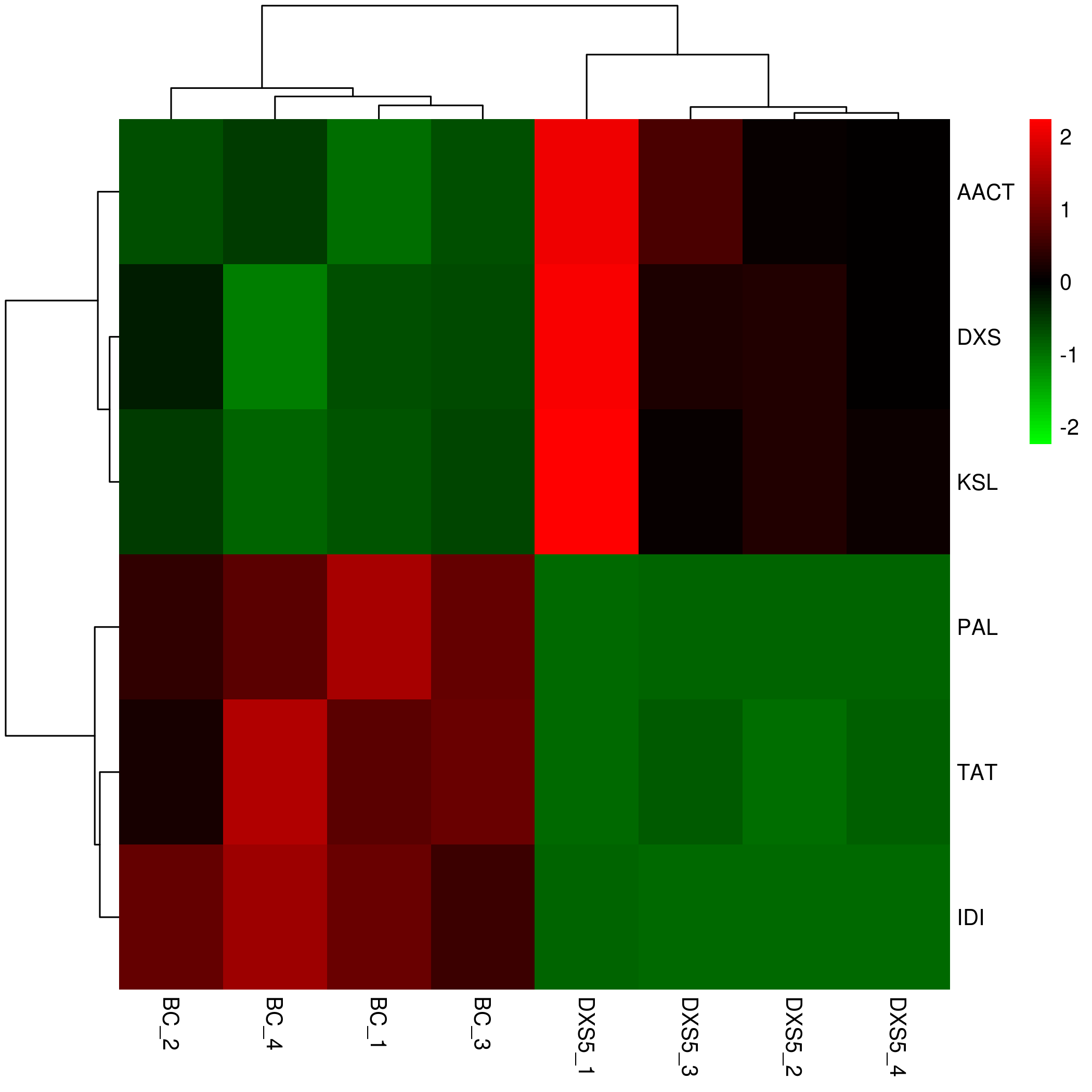

Supplement: Supplementary file 21 [file Image_11.png]
